# Magnetic Actuation for Wireless Capsule Endoscopy in a Large Workspace Using a Mobile-Coil System

**DOI:** 10.3390/mi15111373

**Published:** 2024-11-14

**Authors:** Xiao Li, Detian Zeng, Han Xu, Qi Zhang, Bin Liao

**Affiliations:** 1 School of Electronic Engineering and Automation, Guilin University of Electronic Technology, Guilin 541004, China; lixiao@guet.edu.cn (X.L.); detian0423@163.com (D.Z.); hanxu@mails.guet.edu.cn (H.X.); 2 Key Laboratory of Intelligence Integrated Automation in Guangxi Universities, Guilin 541004, China; 3 School of Mechanical and Electrical Engineering, Guilin University of Electronic Technology, Guilin 541004, China

**Keywords:** WCE, magnetic actuation system, magnetic field modeling, APSO algorithm

## Abstract

Current wireless capsule endoscopy (WCE) is limited in the long examination time and low flexibility since the capsule is passively moved by the natural peristalsis. Efforts have been made to facilitate the active locomotion of WCE using magnetic actuation and localization technologies. This work focuses on the motion control of the robotic capsule under magnetic actuation in a complex gastrointestinal (GI) tract environment in order to improve the efficiency and accuracy of its motion in dynamic, complex environments. Specifically, a magnetic actuation system based on a four-electromagnetic coil module is designed, and a control strategy for the system is proposed. In particular, the proportional–integral–derivative (PID) control parameters and current values are optimized online and in real time using the adaptive particle swarm optimization (APSO) algorithm. In this paper, both simulations and real-world experiments were conducted using acrylic plates with irregular shapes to simulate the GI tract environment for evaluation. The results demonstrate the potential of the proposed control methods to realize the accurate and efficient inspection of the intestine using active WCE. The methods presented in this paper can be integrated with current WCE to improve the diagnostic accuracy and efficiency of the GI tract.

## 1. Introduction

Magnetic actuation technology has extensive applications in microscale robotics and the biomedical field [1,2]. In particular, recently researched magnetically actuated devices, such as capsule endoscopy robots, magnetic catheters, and surgical needles, are being considered for minimally invasive or noninvasive treatments and diagnoses [3,4]. A new type of WCE has been utilized for examining the human GI tract. It is ingested orally to capture images of the GI tract, which doctors use to assess its condition [5]. Compared to traditional endoscopy, WCE can alleviate the pain and discomfort experienced by patients during GI tract examinations [6]. Advancing through the GI tract, the capsule robot efficiently identifies suspicious lesions, reducing the examination time [7]. This solution can minimize medical errors that might occur due to the lack of experience among surgeons and trainees. Given that most clinical operations [8,9] are performed through direct manipulation by clinicians, remote actuation [10,11] is a feasible method for the clinical application of WCE. The WCE requires a magnetic actuation system to generate the magnetic field necessary for remote actuation or other practical functions.

In order to provide the required magnetic field, a magnetic actuation system with multiple fixed electromagnetic coils has been designed and implemented. Helmholtz coils, along with combinations of Helmholtz [12,13] and Maxwell coils [14], are widely used to provide the necessary magnetic force and torque. Compared to fixed systems, mobile magnetic actuation system can generate the required magnetic field over a larger operational area. Magnetic fields are typically generated using either permanent magnets or electromagnetic coils. In mobile systems based on permanent magnets, the magnetic field is adjusted by changing the relative position between the permanent magnet and the capsule endoscope [15]. However, the spatial gradient of the magnetic field is large and unevenly distributed, making the capsule endoscope susceptible to various nonlinear interferences. In contrast, mobile systems based on electromagnetic coils can generate a more controllable magnetic field with the coils being movable and offering a larger operational space.

The essential requirements for a magnetic actuation system include stability, as well as the ability to quickly track desired trajectory signals, suppress disturbances, and avoid oscillations during transient processes. Therefore, careful design of the magnetic actuation system is necessary to achieve better dynamic response. Alireza et al. [16] proposed an adaptive fuzzy sliding mode control method to solve the motion control problem of magnetically actuated microrobots in the presence of input saturation constraint. Huang et al. [17] introduced a model-free control method with a state observer to achieve the path-following control of the team. Zhao [18] proposed a data-driven model predictive control method to control a milli-scale spiral-type magnetic swimmer. Despite the development of many advanced control techniques in the literature, the classic PID controller remains a popular choice due to its simple structure and robustness. However, its gains remain constant during execution, which may reduce the overall performance of the closed-loop system.

To achieve optimal performance, PID parameters must be adjusted and optimized in real time, and many methods have been proposed for this purpose. In addition to the classic Cohen–Coon method, neural networks and artificial intelligence techniques have been utilized. Neural network methods include deep deterministic policy gradient (DDPG) [19], convolutional neural network and long short-term memory (CNN-LSTM) [20], and artificial neural networks (ANNs) [21], while artificial intelligence methods include fuzzy logic [22,23] and intelligent optimization algorithms such as genetic algorithm (GA) [24], particle swarm optimization (PSO) [25], chaos ant colony search [26], artificial bee colony (ABC) algorithm [27], and gravitational search algorithm (GSA) [28], among others, to optimize PID controller parameters.

Although neural networks offer strong adaptability and effective handling of nonlinearities, their limitations in computational resources, data requirements, training complexity, susceptibility to local optima, and interpretability restrict their widespread application. On the other hand, intelligent optimization methods, with their simplicity of implementation, strong global optimization capability, low computational resource requirements, and real-time performance, provide a more flexible and efficient means of solving complex multi-objective optimization problems in control systems. To expand the working space of the magnetic actuation system, the movement of electromagnetic coils is often achieved using a robotic arm as a carrier. By controlling the movement of the robotic arm, the magnetic field can be adjusted, enabling the capsule endoscope to perform targeted inspections and drug delivery tasks. In a system where the magnetic field varies with the spatial position of the robotic arm–electromagnetic coil setup, controlling and optimizing the trajectory of the robotic arm is crucial for improving the uniformity and stability of the magnetic field [29]. Wang et al. [30] proposed an oscillation attenuation control strategy to optimize the joint motion trajectory of the robotic arm, while Wang et al. [31] introduced a variable universe fuzzy control method with an online optimized scaling factor to track the trajectory of the robotic arm.

Aiming to investigate the above problems of the magnetic actuation system and inspired by the above ideas, in this paper, we design a four-electromagnetic coil module that allows the WCE to complete steering at any angle around the origin. The APSO algorithm is employed to optimize the current values required for the WCE to perform real-time, online steering at any angle. Additionally, the motion characteristics of the WCE in the complex environment of the GI tract are studied, and a method is devised for the WCE to perform inspections in square trajectory, infinity trajectory, and circle trajectory patterns based on spatial planarization, simplifying the operational steps for medical personnel. A spatially movable magnetic field platform based on a robotic arm–electromagnetic coil system is constructed, and a mobile electromagnetic coil actuation system based on a PID controller is designed. The APSO algorithm is used to optimize the main parameters in the controller in real time, preventing the parameters from getting trapped in local optima, thereby effectively improving the dynamic response, steady-state performance, and overall control accuracy of the magnetic actuation system.

The following is the arrangement of the rest of this article: In Section 2, the magnetic field model and current calculation equation are derived. In Section 3, the design of the APSO algorithm and controller is described. In Section 4, the structure of the experimental platform is introduced, and the performance of the controller is verified through both simulation and real-world experiments.

## 2. Magnetic Field Modeling and Current Calculation

In this section, a magnetic field model is established and solved orthogonally using the Gauss–Legendre method. Using this method, we derive the current calculation formulas needed for the WCE to complete arbitrary angle steering around the origin based on the magnetic field model. For a single-turn electromagnetic coil, the magnetic field generated at any point in space after a current is applied can be described using the Biot–Savart law as follows:

(1) $$d\vec{B}=\frac{\mu_{0}}{4\pi }\oint_{s}\frac{Id\vec{l}\times \vec{r_{0}}}{|\vec{r_{0}}{|}^{3}}$$

where $Id\vec{l}$ is the element current, $\vec{r_{0}}$ is the vector from the current element on the wire loop to any point (x,y,z), *s* is the infinitely thin path length of the circular wire, and $\mu_{0}=4\pi \times 10^{-7}\;\mathrm{Tm}/A$ is the permeability of free space.

**Figure 1:**
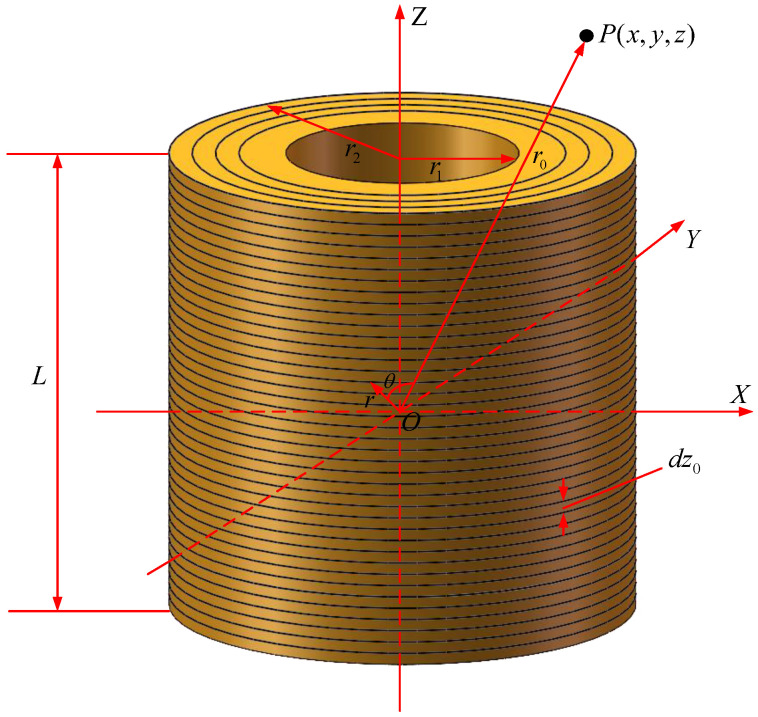
Coil’s structural model whose length and thickness are considered.

The analytical model of the cylindrical electromagnetic coil is based on the Biot–Savart law. To improve the accuracy of the magnetic field description and to extend the effective space where the magnetic field is accurately described, the model in this article takes into account the length and thickness of the electromagnetic coil. The structure of the coil is shown in Figure 1.

As shown in Figure 1, *r* is the radius of the single-turn coil; θ is the angle between radius *r*; and vector $r_{0}$, $r_{1}$ and $r_{2}$ are the inner and outer radius of the coil; *L* is the length of the coil; $dz_{0}$ is the thickness of the single-layer thin coil; and *P* a point in space with coordinates (x,y,z). Substituting the relevant variables into Equation (1), the magnetic field generated by a single-turn coil of length *L* at point *P* is given as follows:

(2) $$\left\{\begin{array}{cc} B_{emx} & =\frac{\mu_{0}N_{R}N_{s}I}{4\pi }\int_{-L/2}^{L/2}dz_{0}\int_{r_{1}}^{r_{2}}dR\int_{0}^{2\pi }\frac{R(z-z_{0})\cos\theta }{{\left[{\left(x-R\cos\theta \right)}^{2}+{\left(y-R\sin\theta \right)}^{2}+{\left(z-z_{0}\right)}^{2}\right]}^{3/2}}d\theta \\ B_{emy} & =\frac{\mu_{0}N_{R}N_{s}I}{4\pi }\int_{-L/2}^{L/2}dz_{0}\int_{r_{1}}^{r_{2}}dR\int_{0}^{2\pi }\frac{R(z-z_{0})\sin\theta }{{\left[{\left(x-R\cos\theta \right)}^{2}+{\left(y-R\sin\theta \right)}^{2}+{\left(z-z_{0}\right)}^{2}\right]}^{3/2}}d\theta \\ B_{emz} & =\frac{\mu_{0}N_{R}N_{s}I}{4\pi }\int_{-L/2}^{L/2}dz_{0}\int_{r_{1}}^{r_{2}}dR\int_{0}^{2\pi }\frac{\left(R^{2}-Rx\cos\theta -Ry\sin\theta \right)}{{\left[{\left(x-R\cos\theta \right)}^{2}+{\left(y-R\sin\theta \right)}^{2}+{\left(z-z_{0}\right)}^{2}\right]}^{3/2}}d\theta \end{array}\right.$$

where $N_{R}$ is the radial coil density, and $N_{s}$ is the axial coil density, which is the number of coils per unit length along the length of the coil.

Since the coil is sufficiently dense, it can be assumed that there are no gaps between them. However, it is very difficult to directly solve Equation (2) through integration. To reduce computational effort, the Gauss–Legendre quadrature method is employed for numerical solution, and the final expression is expressed as follows:

(3) $$\vec{B}=\left(B_{emx},B_{emy},B_{emz}\right)=g\left(x,y,z\right)$$

where $B_{emx}$, $B_{emy}$, and $B_{emz}$ are given as follows:

(4) $$\left\{\begin{array}{cc} k_{1} & =\frac{\mu_{0}N_{R}N_{s}I}{4\pi }\\ B_{emx} & =k_{1}\sum_{i=1}^{n}A_{i}\left[\sum_{j=1}^{n}A_{j}\left(\sum_{k=1}^{n}g_{x}\left(x,y,z,x_{i},x_{j},x_{k}\right)\right)\right]\\ B_{emy} & =k_{1}\sum_{i=1}^{n}A_{i}\left[\sum_{j=1}^{n}A_{j}\left(\sum_{k=1}^{n}g_{y}\left(x,y,z,x_{i},x_{j},x_{k}\right)\right)\right]\\ B_{emz} & =k_{1}\sum_{i=1}^{n}A_{i}\left[\sum_{j=1}^{n}A_{j}\left(\sum_{k=1}^{n}g_{z}\left(x,y,z,x_{i},x_{j},x_{k}\right)\right)\right] \end{array}\right.$$

where $A_{i}$ and $A_{j}$ are Gauss coefficients; *n* is the number of Gauss nodes; $x_{i}$, $x_{j}$, and $x_{k}$ are the Gauss nodes; and the expressions for $g_{x}$, $g_{y}$ and $g_{z}$ are as follows:

(5) $$\left\{\begin{array}{c} T_{2}=\frac{\left(r_{2}-r_{1}\right)t_{2}}{2}+\frac{r_{2}+r_{1}}{2}\\ T_{3}={\left[{\left(x-T_{2}\cos\left(\pi t_{1}+\pi \right)\right)}^{2}+{\left(y-T_{2}\sin\left(\pi t_{1}+\pi \right)\right)}^{2}+{\left(z-\frac{L}{2}t_{3}\right)}^{2}\right]}^{3/2}\\ g_{x}\left(x,y,z,t_{1},t_{2},t_{3}\right)=\frac{T_{2}\left(z-\frac{L}{2}t_{3}\right)\cos\left(\pi t_{1}+\pi \right)}{T_{3}}\\ g_{y}\left(x,y,z,t_{1},t_{2},t_{3}\right)=\frac{T_{2}\left(z-\frac{L}{2}t_{3}\right)\sin\left(\pi t_{1}+\pi \right)}{T_{3}}\\ g_{z}\left(x,y,z,t_{1},t_{2},t_{3}\right)=\frac{T_{2}^{2}-T_{2}x\cos\left(\pi t_{2}+\pi \right)-T_{2}y\sin\left(\pi t_{1}+\pi \right)}{T_{3}} \end{array}\right.$$

**Figure 2:**
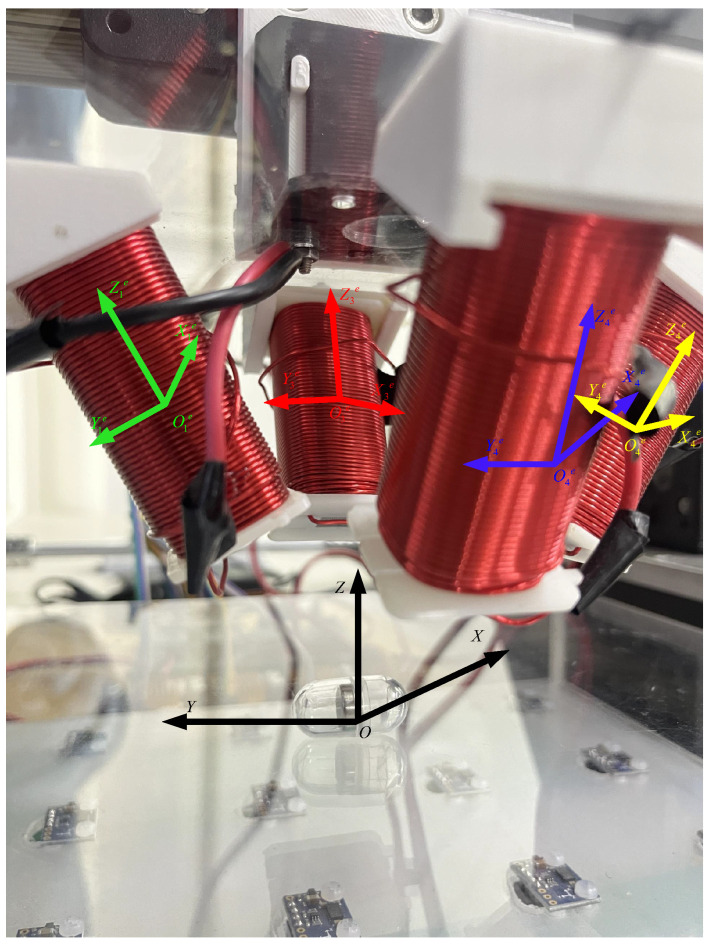
Diagram of the four-electromagnetic coil coordinate system.

This paper involves the design of a four-electromagnetic coil module to control the WCE in completing arbitrary angle steering around the origin. The coil numbering and coordinate system are shown in Figure 2. Assuming that there is an arbitrary point D(x,y,z) in the global coordinate system O−XYZ, a local coordinate system $O_{i}^{e}-X_{i}^{e}Y_{i}^{e}Z_{i}^{e}$ is established for each coil, with the center of each coil as the origin.

An arbitrary point D(x,y,z) in the global coordinate system can be transformed into the coil coordinate system, where $D_{i}^{\prime }\left(i=1,2,3,4\right)$ represents the coordinates of point D(x,y,z) in the coordinate system of each coil. The $\theta_{i}\left(i=1,2,3,4\right)$ values represent the tilt angles of the four coils relative to the mounting plane, with $\theta_{1}=\theta_{4}=-30^{\circ }$ and $\theta_{2}=\theta_{3}=30^{\circ }$, where *i* denotes the coil index. The resultant magnetic field at the point produced by the four coils can then be calculated using the magnetic field calculation formula for electromagnetic coils given in Equation (6).

(6) $$\left\{\begin{array}{cc} B_{x} & =\sum_{i=3}^{4}I_{i}f_{em}\left(D_{i}^{\prime }\right)\sin\theta_{i}\\ B_{y} & =\sum_{i=1}^{2}I_{i}f_{em}\left(D_{i}^{\prime }\right)\sin\theta_{i}\\ B_{z} & =\sum_{i=1}^{4}I_{i}f_{em}\left(D_{i}^{\prime }\right)\cos\theta_{i} \end{array}\right.$$

Here, $f_{em}\left(x,y,z\right)$ is the result obtained by substituting the coordinate values into Equation (4) for calculation.

Because the position of the WCE changes every moment, the expected magnetic field changes every moment as well; when the desired magnetic field at the target point is $\left(B_{x}^{*},B_{y}^{*},B_{z}^{*}\right)$, the following equation is satisfied:

(7) $$\left(B_{x}^{*},B_{y}^{*},B_{z}^{*}\right)=\left(\begin{array}{cccc} 0 & 0 & f_{em}\left(D_{3}^{\prime }\right)\sin\theta_{3} & f_{em}\left(D_{4}^{\prime }\right)\sin\theta_{4}\\ f_{em}\left(D_{1}^{\prime }\right)\sin\theta_{1} & f_{em}\left(D_{2}^{\prime }\right)\sin\theta_{2} & 0 & 0\\ f_{em}\left(D_{1}^{\prime }\right)\cos\theta_{1} & f_{em}\left(D_{2}^{\prime }\right)\cos\theta_{2} & f_{em}\left(D_{3}^{\prime }\right)\cos\theta_{3} & f_{em}\left(D_{4}^{\prime }\right)\cos\theta_{4} \end{array}\right)\left(\begin{array}{c} I_{1}\\ I_{2}\\ I_{3}\\ I_{4} \end{array}\right)$$

To determine the relationship between the desired magnetic field and the currents through each coil, we need to solve this equation. However, since this is an underdetermined system, with four unknowns but only three equations, it is impossible to find a unique solution. To address this, we employ an optimization algorithm, where the conditions above are treated as constraints. The objective function is defined as follows:

(8) $$\begin{array}{cc} \; & \min\;F=\left(\right|I_{1}|-I_{a}{)}^{2}+\left(\right|I_{2}|-I_{a}{)}^{2}+\left(\right|I_{3}|-I_{a}{)}^{2}+\left(\right|I_{4}{\left|-I_{a}\right)}^{2}\\ \; & s.t.\;0<I_{i}<1 \end{array}$$

where $I_{i}$ represents the currents of four coils (i=1,2,3,4), and $I_{a}$ represents the control current obtained in the experiment, with $I_{a}=0.3A$.

Assuming the WCE remains at the central control point *O*, and due to the geometric symmetry of the system, the distances from each coil to the central control point are equal. Under this symmetric configuration, the contribution of each coil to the magnetic field can be approximated as equal. Therefore, Equation (9) simplifies to the following form:

(9) $$f_{em}\left(D_{1}^{\prime }\right)=f_{em}\left(D_{2}^{\prime }\right)=f_{em}\left(D_{3}^{\prime }\right)=f_{em}\left(D_{4}^{\prime }\right)=p$$

This symmetry assumption greatly simplifies the calculation of the magnetic field contributions, allowing the contribution from each coil to be represented by the same constant *p*, thus simplifying the computation process.

Then, the Equation (7) can be transformed into the following:

(10) $$\left(B_{x}^{*},B_{y}^{*},B_{z}^{*}\right)=\left(\begin{array}{cccc} 0 & 0 & \frac{1}{2}p & -\frac{1}{2}p\\ -\frac{1}{2}p & \frac{1}{2}p & 0 & 0\\ \frac{\sqrt{3}}{2}p & \frac{\sqrt{3}}{2}p & \frac{\sqrt{3}}{2}p & \frac{\sqrt{3}}{2}p \end{array}\right)\left(\begin{array}{c} I_{1}\\ I_{2}\\ I_{3}\\ I_{4} \end{array}\right).$$

The relationship between the reverse-solved current and the magnetic field is presented below, and the current for the four actuating coils can be expressed as follows:

(11) $$\left\{\begin{array}{cc} I_{1} & =I_{D}\\ I_{2} & =\frac{2}{p}B_{y}^{*}+I_{D}\\ I_{3} & =\frac{\sqrt{3}}{3p}B_{z}^{*}+\frac{1}{p}B_{x}^{*}-\frac{1}{p}B_{y}^{*}-I_{D}\\ I_{4} & =\frac{\sqrt{3}}{3p}B_{z}^{*}-\frac{1}{p}B_{x}^{*}-\frac{1}{p}B_{y}^{*}-I_{D} \end{array}\right.$$

In Equation (11), the currents $I_{1}$, $I_{2}$, $I_{3}$, and $I_{4}$ are the control variables. By adjusting these currents, different magnetic fields can be generated. The reference current $I_{D}$ serves as the excitation current, defined as $I_{D}=I_{1}$. The desired magnetic field components $B_{x}^{*}$, $B_{y}^{*}$, and $B_{z}^{*}$ represent the state variables and outputs of the system. These state variables describe the magnetic field distribution at a specific moment. By fine-tuning the currents, the system induces corresponding changes in the magnetic field, thus controlling the orientation of the WCE.

## 3. Optimization Algorithm and Controller Design

In this section, the control parameters of the system, including the current values for the four coils and the X and Y coordinates of the robotic arm, are determined to ensure the precise control of the WCE.

**Figure 3:**
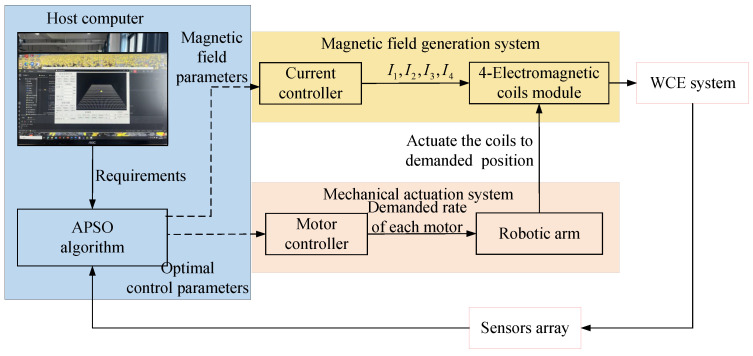
The system’s schematic diagram.

The system designed in this paper consists of four main structures: host computer, magnetic field generation system, mechanical actuation system, and localization devices. In this design, the calculation process is distributed to different subsystems to reduce mutual interference and increase speed. The localization of the WCE [32], the values of the four coil currents, and optimization on coils’ positions are calculated in the host computer. The optimal parameters for the coils’ positions are sent to the mechanical actuation system for further processing. The angles of each motor are calculated by the motor controller and then executed by the robot arm. The current values required to generate the magnetic field for the WCE to complete its steering are sent to the magnetic field generation system, where the current controller produces the necessary current based on the specified angles. A schematic diagram of the system is shown in Figure 3.

The values of the currents for the four coils are optimized using the APSO algorithm during each experiment to ensure that the WCE achieves optimal performance during directional changes. The rotational angle of the WCE is defined as the angle between the north pole of the permanent magnet inside the capsule and the positive X-axis of the global coordinate system. First, the host computer sets the desired steering angles based on the target trajectory of the WCE. These steering angles are then input into the APSO algorithm, which dynamically computes the optimal current values for each coil. The APSO algorithm iterates current values and evaluates them based on how well they generate the magnetic field required for the specified angles. Eventually, the algorithm converges to the current values that best match the target steering angles. These optimized current values are then applied to the coils in real time, enabling the system to adjust the steering direction of the WCE with high precision and stability.

The X and Y coordinates of the robotic arm are precisely controlled with a PID controller to maintain accurate control of the WCE. Based on real-time feedback from the WCE system, the PID controller continuously adjusts the position of the robotic arm, ensuring precise corrections to accurately track the target trajectory.

### 3.1. APSO Algorithm

The PSO algorithm [33] is a stochastic search algorithm inspired by the hunting behavior of bird flocks. In the PSO algorithm, the update equation for the *i* th particle at the *k* th iteration is given as follows:

(12) $$\left\{\begin{array}{cc} V_{i}\left(k+1\right) & =\omega V_{i}\left(k\right)+c_{1}r_{1}\left[P_{i}\left(k\right)-X_{i}\left(k\right)\right]+c_{2}r_{2}\left[P_{g}\left(k\right)-X_{i}\left(k\right)\right]\\ X_{i}\left(k+1\right) & =X_{i}\left(k\right)+V_{i}\left(k+1\right),\;i=1,2,\cdots ,m \end{array}\right.$$

In Equation (12), ω is the inertia weight, $c_{1}$ and $c_{2}$ are the learning factors, and $r_{1}$ and $r_{2}$ are random numbers uniformly distributed in the range [0, 1]. $P_{i}=\left(p_{i1},p_{i2},\cdots ,p_{id}\right)$ represents the best position of the individual, and $P_{g}=\left(p_{g1},p_{g2},\cdots ,p_{gd}\right)$ represents the global best position. However, the standard PSO algorithm often struggles to effectively balance global and local optima.

**Figure 4:**
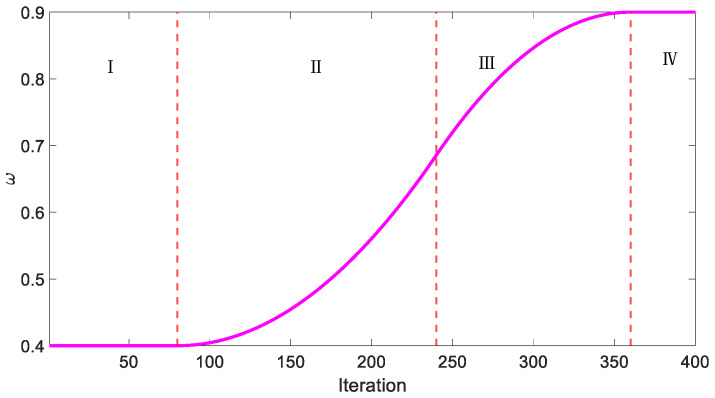
The curve shows the variation of ω with the iteration number.

Therefore, this article proposes the APSO algorithm that increases the inertia weight nonlinearly. The expression is expressed as follows:

(13) $$\omega =\left\{\begin{array}{cc} \omega_{\min} & k\leq a\\ \omega_{\min}+\left(\omega_{\max}-\omega_{\min}\right)*\frac{{\left(k-a\right)}^{2}}{\left(c-a)(b-a\right)} & a<k\leq b\\ \omega_{\max}-\left(\omega_{\max}-\omega_{\min}\right)*\frac{{\left(c-k\right)}^{2}}{\left(c-a)(c-b\right)} & b<k\leq c\\ \omega_{\max} & k>c \end{array}\right.$$

where *k* is the iteration number, and *a*, *b*, and *c* satisfy $\omega_{\min}<a\leq b\leq c<\omega_{\max}$. In this case, $a=0.2k_{\max}$, $b=0.6k_{\max}$, and $c=0.9k_{\max}$. Assuming the maximum number of iterations $k_{\max}=400$, Figure 4 shows the variation curves of different inertia weights ω.

Figure 4 shows how the inertia weight ω changes as the number of iterations increases, broken down into four distinct phases, with each phase corresponding to specific iteration intervals. In region I, ω remains constant at its minimum value $\omega_{\min}$. At the start of the optimization process, keeping ω low encourages the algorithm to explore a wide area of the search space, reducing the likelihood of early convergence to suboptimal solutions. In region II, as the number of iterations increases, ω starts to rise nonlinearly. This gradual increase in ω accelerates the convergence process by focusing the search more narrowly, increasing the chances of identifying regions near the global optimum. In region III, ω continues to increase as the iteration count grows. This stage shifts the focus from global exploration to local exploitation, enhancing local search capabilities while avoiding premature convergence. The search speed decreases as ω increases, allowing for more fine-grained searches. In region IV, ω reaches its maximum value $\omega_{\max}$ and stabilizes. In this final phase, the constant $\omega_{\max}$ value enables precise fine-tuning around the optimal solution, ensuring that the search converges smoothly and accurately.

Through the dynamic adjustment of ω based on iteration count, the APSO algorithm is able to balance global exploration and local exploitation. This ensures that the algorithm avoids premature convergence and fine-tunes the solution in the later stages, leading to both effective and precise optimization.

As indicated by Equation (11), there is a complex coupling relationship between the four current values. This implies that during the optimization process, any change in one current value will trigger a chain reaction affecting the other current values, thereby increasing the complexity of the optimization problem. The traditional PSO algorithm is typically only capable of handling simple optimization problems. When faced with such highly coupled systems, their fixed inertia weight and lack of dynamic adjustment often result in premature convergence to local optima, leading to suboptimal optimization outcomes. In contrast, the APSO algorithm dynamically adjusts the inertia weight in real time, achieving a better balance between global and local searches, which makes it particularly suitable for handling complex optimization problems with coupled relationships. This significantly increases the likelihood of escaping local optima. Therefore, when dealing with optimization problems involving complex coupling relationships, the APSO algorithm not only better addresses the coupling between current values but also ensures that the optimization results for the currents align more closely with expectations, thereby improving the overall performance of the system. This demonstrates the APSO algorithm’s greater adaptability and superiority in solving complex, multivariable optimization problems.

### 3.2. Motor Controller Design

To ensure the WCE operates stably within the human GI tract, precise control is essential to reduce internal viscous resistance and avoid external disturbances. The actuation control of the WCE involves managing several complex variables, including speed control in the narrow and curved sections of the GI tract and the ability to adapt to external disturbances. Although some advanced control methods, such as signal control [34], can achieve high precision under certain conditions, they often rely on idealized hardware setups to deliver optimal performance. Given the complexity of physical environments, we adopt the widely validated PID controller, which is known for its low hardware requirements, simple structure, and reliability. The PID controller delivers consistent control in dynamic and complex environments, ensuring that the system meets performance expectations across various real-world applications.

However, the traditional PID controller, with its fixed parameters, often lacks adaptability in complex dynamic environments, making it difficult to adjust parameters effectively and handle nonlinear system characteristics or varying external disturbances.

**Figure 5:**
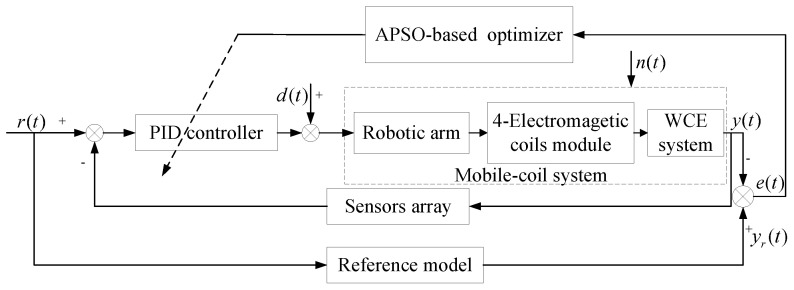
APSO-PID control system’s block diagram.

These limitations prevent traditional PID controllers from delivering optimal control in complex vivo conditions. To address these challenges, this study utilizes the APSO algorithm to dynamically optimize the six PID control parameters, enabling the WCE to swiftly and accurately follow desired trajectory signals. This approach enhances the safety and effectiveness of GI tract inspection. The APSO-PID actuation system block diagram is shown in Figure 5.

In the mobile-coil system illustrated in Figure 5, the robotic arm is coordinated by X- and Y-axis motors, ensuring that the WCE can swiftly and accurately track various inspection paths. To navigate the complex environment and unpredictable external disturbances in the GI tract, this study utilizes a discrete positional PID controller. This controller effectively captures the difference between the current state and the target state by accumulating previous errors through its integral term, thus enabling precise control. The discrete positional PID controller, with its unique algorithmic design, allows the magnetic actuation system to respond rapidly, adapting nimbly to the complex internal environment of the body. It achieves high control precision, which is crucial for tasks such as inspecting small lesions, while also delivering robust steady-state performance. This ensures that the system remains stable during prolonged operation within the body, unaffected by dynamic physiological changes. The formulation of the controller is as follows:

(14) $$u_{i}\left(k\right)=K_{p_{i}}e_{i}\left(k\right)+K_{i_{i}}\sum_{k=1}^{n}e_{i}\left(k\right)T+K_{d_{i}}\frac{e_{i}\left(k\right)-e_{i}\left(k-1\right)}{T}$$

where *T* is the time step; when *i* is X or Y, $u_{i}\left(k\right)$ represents the control input for the X- or Y-axis motor, which adjusts the rotation speed of the corresponding motor to control the position of the WCE along the X or Y axis; $e_{i}\left(k\right)$ represents the difference between the desired position produced by the expected model on the X or Y axis and the actual position of the WCE, the error function is expressed as follows:

(15) $$e_{i}\left(k\right)=y_{r_{i}}\left(k\right)-y_{i}\left(k\right)$$

The smaller the error, the closer the controlled system is to the ideal desired model. Therefore, the performance index J can be used to evaluate the controller.

(16) $$J\left(K_{p_{i}},K_{i_{i}},K_{d_{i}}\right)=\int_{0}^{t}t{\left[y_{r_{i}}\left(t\right)-y_{i}\left(t\right)\right]}^{2}d\tau$$

**Figure 6:**
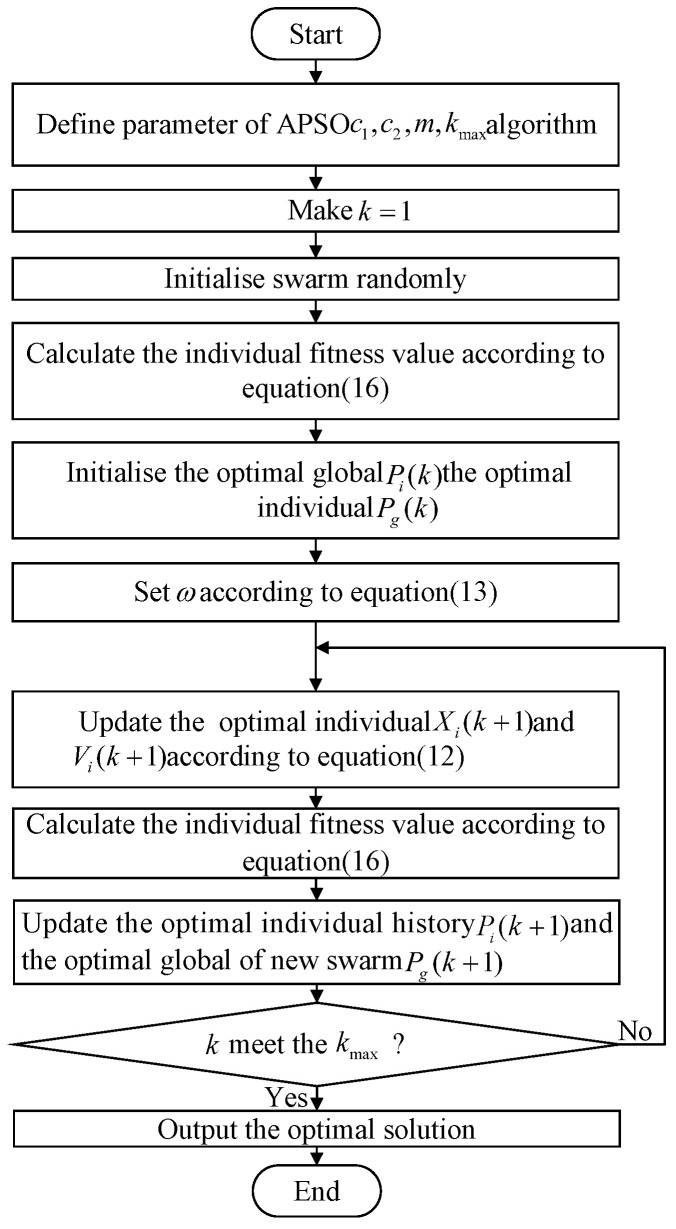
Flowchart of APSO algorithm.

The above equation illustrates the degree of proximity between the controlled system and the ideal desired model, including $K_{p_{x}}$, $K_{p_{y}}$, $K_{i_{x}}$, $K_{i_{y}}$, $K_{d_{x}}$, and $K_{d_{y}}$, which represent the six adjustable parameters. These six adjustable parameters are closely interrelated, leading to strong coupling among them. The complexity and nonlinearity of the system, along with the tedious and uncertain nature of manual tuning and the inadequate adaptive response in dynamic environments, make it difficult for traditional fixed-parameter PID control to achieve optimal results. To address these challenges, this paper introduces the APSO algorithm to dynamically optimize the PID controller parameters. The APSO-PID parameter optimization problem is thus framed as a multi-objective optimization problem, as expressed in the following equation:

(17) $$\left(K_{p_{i}}^{*},K_{i_{i}}^{*},K_{d_{i}}^{*}\right)=\arg\;\min J\left(K_{p_{i}},K_{i_{i}},K_{d_{i}}\right)$$

where $K_{p_{x}}^{*}$, $K_{p_{y}}^{*}$, $K_{i_{x}}^{*}$, $K_{i_{y}}^{*}$, $K_{d_{x}}^{*}$, and $K_{d_{y}}^{*}$ represent the PID parameters optimized by the APSO algorithm. The flow of the algorithm is shown in Figure 6.

## 4. Results and Analysis

A cylindrical permanent magnet was mounted in the WCE to control its movement by actuating the magnet. Three different controllers were used in both system simulations and real-world experiments to validate the performance of the designed controllers.

To achieve optimal control performance, we conducted multiple simulation experiments using a predefined trajectory with disturbances. During these simulations, the results were used as inputs for the optimization algorithms, allowing for parameter tuning based on the response of the system to each trajectory. The final optimized PID parameters obtained from this process were then applied consistently across all trajectory tracking tasks presented in this study. This approach ensures that the control methods are evaluated under uniform conditions, providing a reliable basis for comparing their performance.

### 4.1. Simulation Results and Analysis

To evaluate the control performance of the APSO-PID controller and validate the effectiveness of the APSO algorithm, comparisons were conducted with PSO-PID and ZN-PID [35] controllers. ZN-PID refers to a PID controller whose parameters are tuned using the Ziegler–Nichols method. This classical method is one of the most widely used techniques for determining the proportional, integral, and derivative gains of a PID controller. The Ziegler–Nichols method provides a systematic way to set the controller parameters based on the response of the system to a step input, aiming for a balance between stability and responsiveness. The PID parameters were optimized separately using three distinct methods including ZN, PSO, and APSO algorithms, according to the degree of deviation of the WCE from its desired trajectory. In each experiment, ZN, PSO, and APSO algorithms were individually applied to optimize the PID parameters, thus enabling a comparative analysis of their effectiveness in maintaining accurate control. ZN and PSO algorithms were used for static parameter optimization, while the APSO algorithm dynamically adjusted the parameters in response to trajectory changes, allowing for a comprehensive evaluation of the performance of each method across different trajectories. The parameter settings for the APSO-PID controller are detailed in Table 1.

In order to prevent the WCE from failing to accurately track the predefined trajectory at the inflection points of the square, infinity, and circular trajectories, and to reduce system response lag, the range of the three PID parameters was determined after several simulations and experiments, as follows: $45\leq K_{p}\leq 75$, $15\leq K_{i}\leq 29$ and $0\leq K_{d}\leq 2$.

**Figure 7:**
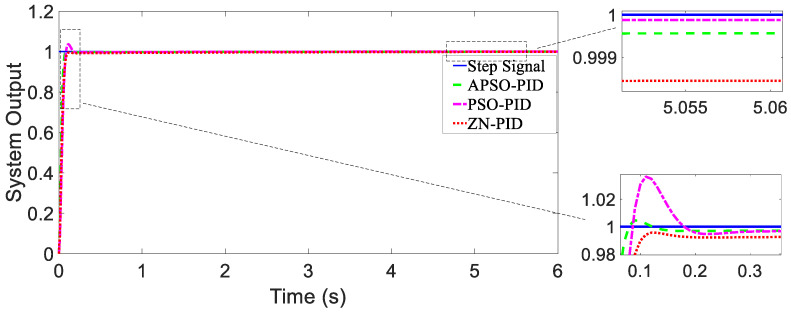
The step response of the magnetic actuation system with different controllers (no disturbance).

When there are no external disturbances in the system, the unit step response curves of the magnetic actuation system using different controllers were plotted, which are shown in Figure 7.

In Figure 7, the system output represents a combined metric that includes both the orientation of the WCE and a calculated value based on its X and Y positional coordinates.

**Figure 8:**
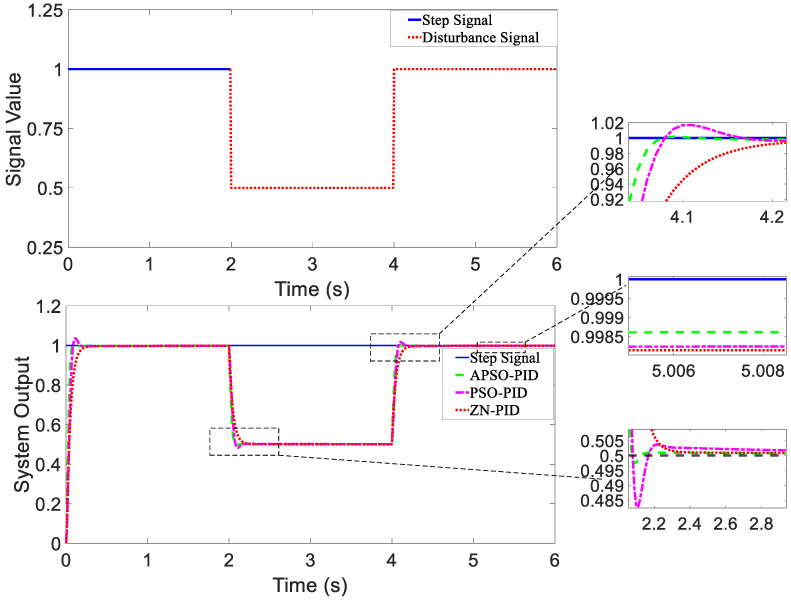
Composite disturbance signal and controller response curves.

For the purpose of evaluating the dynamic response performance of different controllers under disturbance, two types of disturbance signals were introduced at the signal input for simulation testing. Disturbances were introduced at the control inputs of the X and Y motors, which led to changes in motor speed and subsequently influenced the position of the WCE. The first disturbance signal was a composite disturbance consisting of two parts. A downward step disturbance was applied at 2 s, followed by an upward step disturbance at 4 s. The input disturbance signal and the response curves of the three controllers are shown in Figure 8.

**Figure 9:**
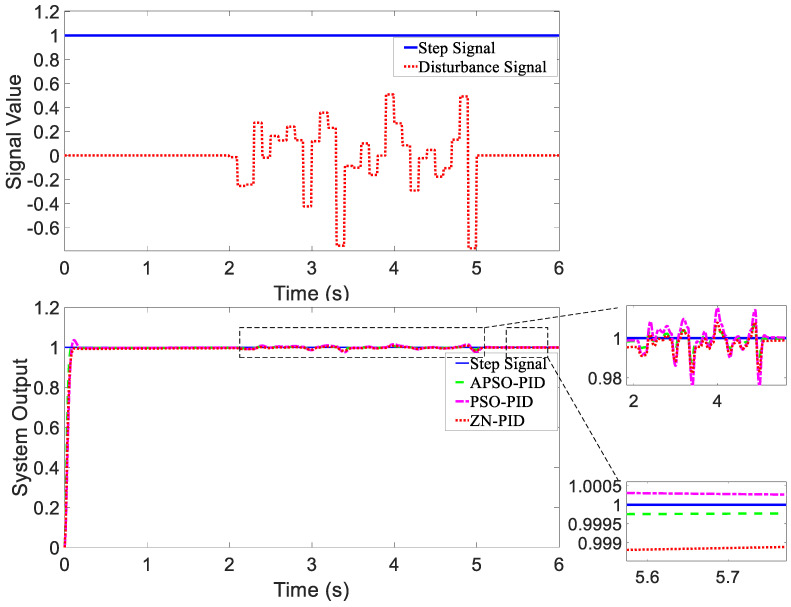
Random disturbance signal and controller response curves.

The second disturbance signal was a random disturbance. Between 2 and 5 s, a random disturbance signal was applied to the controller. The form of the random disturbance signal and the output response curves of the three controllers are shown in Figure 9.

In Figure 8 and Figure 9, the system output reflects a combined metric that takes into account both a calculated value based on its X and Y positional coordinates and the orientation of the WCE.

Table 2 provides a comparison of the performance of the three controllers in the magnetic control system, under conditions with no disturbance as well as two different types of disturbances.

(1) Analysis of Tracking Responsiveness
  As shown in Figure 7, when there were no external disturbances, all three controllers exhibited good dynamic response performance, with each achieving the desired minimal overshoot. The tracking performance of the three controllers was similar. As shown in Table 2, compared to the ZN-PID and PSO-PID controllers, the setting time of the APSO-PID controller was reduced by 67.6% and 25.3%, respectively, indicating that the APSO-PID controller significantly outperformed the other two in terms of speed.
(2) Analysis of Anti-disturbance Performance
  Figure 8 and Figure 9 show that the APSO-PID controller exhibited greater robustness under different disturbance inputs compared to the other two controllers. As shown in Table 2, under the influence of the composite disturbance signal, the APSO-PID controller reduced the setting time by 63.5% and 71.8% relative to the ZN-PID and PSO-PID controllers, respectively. Similarly, under the influence of the random disturbance signal, the setting time was reduced by 59.9% and 61.6% compared to the ZN-PID and PSO-PID controllers, respectively. This indicates that the APSO-PID controller has the best anti-disturbance performance, followed by the PSO-PID and ZN-PID controllers.
(3) Analysis of Steady-State Performance
  As shown in Table 2, all three controllers demonstrated strong steady-state performance, with steady-state error approaching 0. Considering both convergence speed and overshoot into account, the APSO-PID controller performed the best overall. As shown in Figure 8, the APSO-PID and PSO-PID controllers maintained solid steady-state performance after the disturbance signal was introduced, quickly returning to a steady state with minimal steady-state error. The APSO-PID controller yielded the smallest error. In contrast, the ZN-PID controller showed weaker steady-state performance, with significant oscillations and longer recovery times due to its higher sensitivity to disturbances. Figure 9 shows that under random disturbance signals, the APSO-PID and PSO-PID controllers continued to perform well, with almost negligible steady-state errors. However, the ZN-PID controller fell short, displaying slight deviations compared to the other two controllers.

Overall, the APSO-PID controller outperformed the other two controllers. Although the ZN-PID controller did not exhibit any overshoot after tuning, its rise time was slower, and its dynamic response and setting time were suboptimal when disturbances were present, resulting in poor disturbance rejection performance. The PSO-PID controller demonstrated good dynamic performance but had a larger overshoot. The APSO-PID controller achieved the fastest rise and set times and exhibited the best disturbance rejection performance among the three controllers.

Given the complex environment of the human GI tract, additional testing of controller performance is essential to optimize the control of WCE during inspections and targeted drug delivery. Following the unit step tests, simulations were carried out using square trajectory, infinity trajectory, and circular trajectory to further evaluate their dynamic performance, steady-state accuracy, and anti-disturbance capabilities.

**Figure 10:**
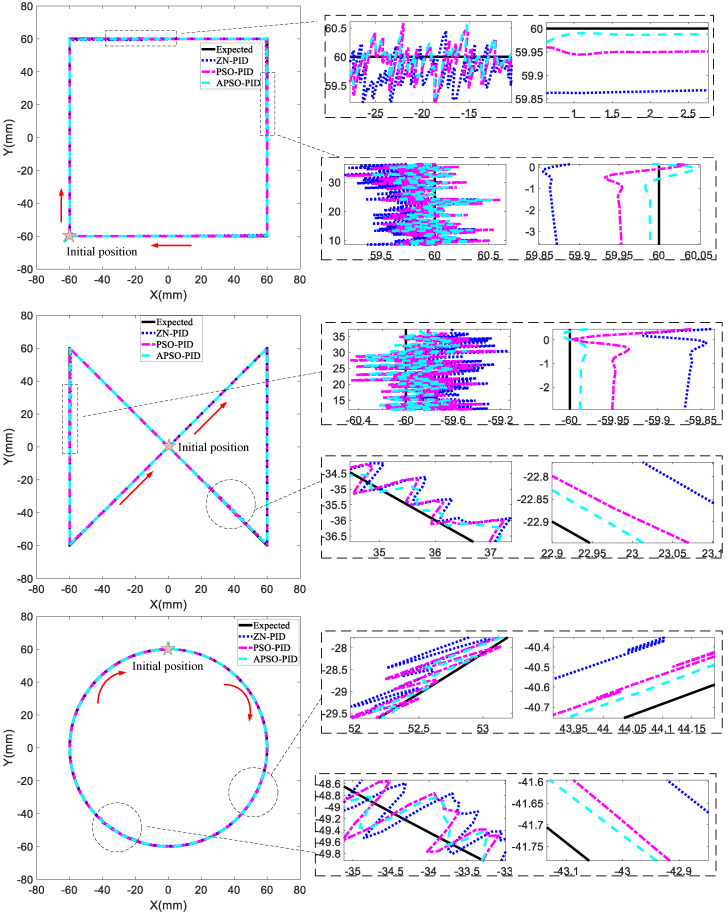
Simulation results of the composite disturbance.

Figure 10 shows the response of the three controllers to two segments of random disturbance signals applied during the square trajectory, infinity trajectory, and circular trajectory. The disturbance signals applied during each trajectory were introduced as controlled external perturbations to the input signals of the X and Y motors. These perturbations simulated external environmental factors, enabling a realistic assessment of how well each controller can maintain stability and accurate tracking under variable conditions. The disturbance periods for the square trajectory were 14–18 s and 26–31 s; for the infinity trajectory, they were 27–31 s and 56–59 s; and for the circular trajectory, they were 21–25 s and 42–45 s. It can be seen from the figures that there is some initial jitter, which is due to the rigid mechanical structure of the magnetic actuation system overcoming friction at startup. The left side of the zoomed figures presents the disturbance rejection performance of the three controllers during the initial phase of disturbance introduction, while the right side displays their tracking performance after the disturbance stabilized. Among the three trajectories, the APSO-PID controller, despite exhibiting slightly larger overshoot at certain moments, generally demonstrated excellent dynamic performance, with shorter regulation times, faster recovery after disturbances, and superior robustness compared to the other two controllers. It also achieved the smallest tracking error.

After evaluating the performance of the three controllers in tracking the trajectories shown in Figure 10, the overall conformity between the desired and actual trajectories was assessed using RMSE and MAE. RMSE is defined as the square root of the average of the squared differences between the actual and desired values across the sample size *N*. MAE is defined as the average of the absolute differences between the actual and desired values. The calculation formulas for RMSE and MAE are given in Equations (18) and (19).

(18) $$RMSE=\sqrt{\frac{1}{N}\sum_{i=1}^{N}\left[{\left(x_{i}-x_{di}\right)}^{2}+{\left(y_{i}-y_{di}\right)}^{2}\right]}$$

(19) $$MAE=\frac{\sum_{i=1}^{N}\left[|x_{i}-x_{di}\left|+\right|y_{i}-y_{di}|\right]}{N}$$

where *N* is the sample size, $x_{i}$ and $y_{i}$ are the coordinates of the *i* th actual trajectory point, and $x_{di}$ and $y_{di}$ are the coordinates of the *i* th desired trajectory point.

Table 3 summarizes the RMSE and MAE for the three controllers when tracking the three trajectories. The data show that, for the square trajectory, the APSO-PID controller improved control accuracy by 0.23 mm compared to the ZN-PID controller and 0.09 mm compared to the PSO-PID controller. For the infinity trajectory, the APSO-PID controller achieved a precision increase of 0.19 mm over the ZN-PID controller and 0.09 mm over the PSO-PID controller. In the circular trajectory, the APSO-PID controller similarly enhanced accuracy by 0.19 mm compared to the ZN-PID controller and 0.06 mm compared to the PSO-PID controller. These results clearly demonstrate that the APSO-PID controller delivers the best performance, with the PSO-PID controller ranking second, and the ZN-PID controller performing the least effectively.

**Figure 11:**
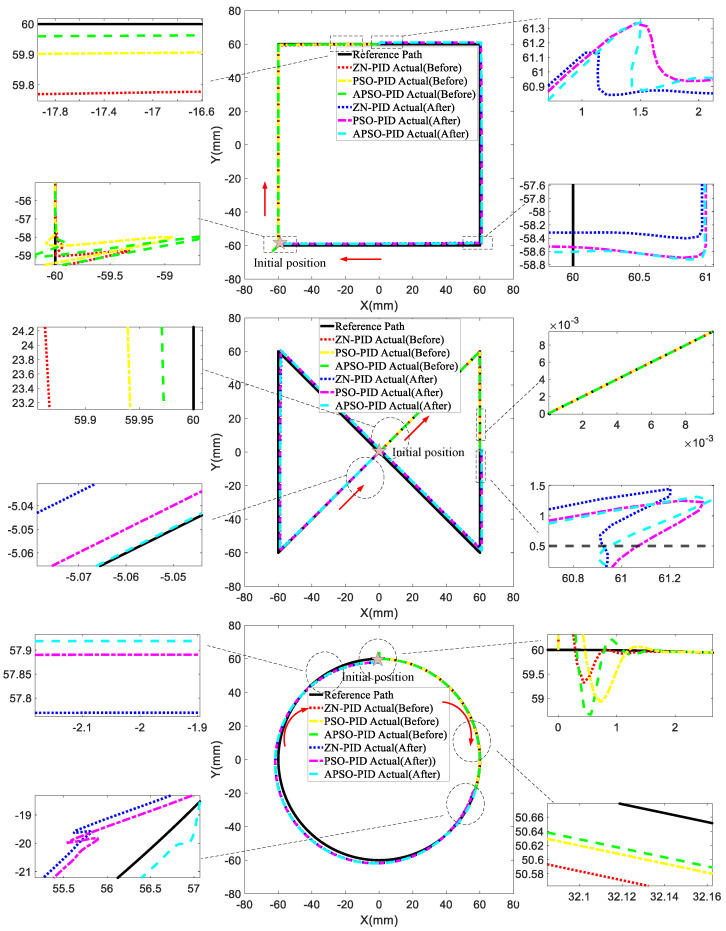
Simulation results for upward step disturbance.

To further evaluate the tracking performance of the three controllers, an upward step signal was introduced as a disturbance at the input. This step signal had a magnitude of +0.5 units and was applied in the upward direction along both the X and Y axes. This approach allowed us to evaluate the robustness of the system in maintaining stability and tracking accuracy under abrupt disturbances. For the square trajectory, the disturbance began at 18 s and continued until the end; for the infinity trajectory, it started at 20 s and persisted until the end; and for the circular trajectory, the disturbance started at 23 s and lasted until the end. The tracking performance of the controllers across these three trajectories was observed. As shown in Figure 11, prior to the disturbance, the APSO-PID controller exhibited a slightly larger overshoot during the initial phase of the square trajectory and circular trajectory compared to the other two controllers. However, the duration of this overshoot was brief, and the controller quickly adjusted to accurately track the desired trajectory.

Among the three trajectories, the APSO-PID controller yielded the smallest tracking error and demonstrated superior trajectory-following capability. During the disturbance, for the square and infinity trajectories, the subgraphs indicate that the APSO-PID controller experienced slightly more overshoot compared to the other two controllers. However, it quickly regained the desired trajectory immediately after the disturbance. The ZN-PID controller showed the fastest setting time and the least overshoot in the subgraphs, which can be attributed to its poor pre-disturbance tracking performance, resulting in a response that closely mirrored the disturbance signal. While this gave the appearance of better performance during the disturbance, the comparison in Table 4 reveals that the overall tracking performance of the ZN-PID controller was inferior to that of the PSO-PID and APSO-PID controllers. The APSO-PID controller demonstrated the best dynamic performance and robustness. For the circular trajectory, the APSO-PID controller stood out with its shorter setting time, minimal overshoot, and greater robustness. As shown in Table 4, after the disturbance, the APSO-PID controller improved control accuracy by 0.13 mm and 0.07 mm on the square trajectory compared to the ZN-PID and PSO-PID controllers, respectively; by 0.10 mm and 0.04 mm on the infinity trajectory; and by 0.03 mm and 0.02 mm on the circular trajectory.

**Figure 12:**
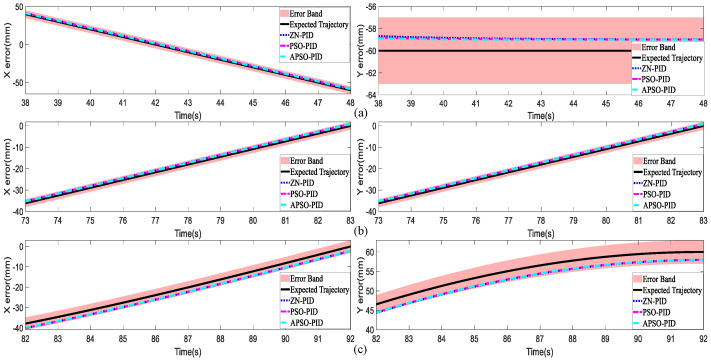
Steady-state performance of the three trajectories. (**a**) Error bands and tracking curves for the square trajectory along the X and Y axes; (**b**) Error bands and tracking curves for the infinity trajectory along the X and Y axes; (**c**) Error bands and tracking curves for the circular trajectory along the X and Y axes.

Following the assessment of dynamic performance, the steady-state performance of the controllers was further inspected. A 5% error band was set to assess whether the three controllers could maintain the trajectory within this range during the final 10 s of tracking. Figure 12a–c illustrate the error bands and the tracking curves for the square trajectory, infinity trajectory, and circular trajectory along the X and Y axes. As shown in the figures, all three controllers successfully remained within the 5% error band across the three trajectories, demonstrating solid steady-state performance. The APSO-PID controller consistently tracked closer to the desired trajectory in all cases, showing superior steady-state performance compared to the other two controllers. Overall, the APSO-PID controller excelled in both dynamic and steady-state performance, outperforming the other two controllers.

### 4.2. Actual Experimental Analysis

**Figure 13:**
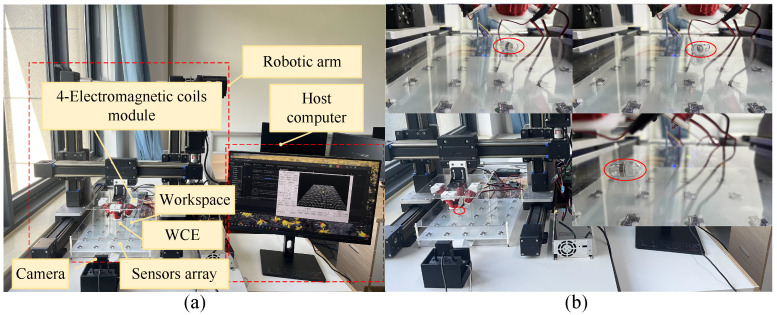
(**a**) Experimental setup of the magnetic actuation system; (**b**) inspection routes followed by the WCE during different trajectory inspections, with the WCE position highlighted in red circles.

Figure 13a shows the experimental setup of the magnetic actuation system proposed in this paper for use in the human GI tract. The system features a three-axis gantry robot arm that controls the movement of an electromagnetic coil mounted on it. The robot was actuated by two motors (86BYGH80, Leadshine, Guangzhou, China) that coordinated movement along the X and Y axes. The electromagnetic coil was constructed with approximately 300 ± 10 turns of 1mm diameter copper wire, wound around a silicon steel core to enhance the magnetic field. The WCE (12.7 mm in diameter and 25.3 mm in length) was composed of a shell (polyethylene terephthalate (PET)) and a permanent magnet (NdFeB42, WANGCl CIYE, Shanghai, China, 10 mm in diameter and 5 mm in length). A large external sensor array, comprising 25 triaxial sensors (LSM303D, ElecFans, Shenzhen, China),was arranged in a 5 × 5 grid with 60 mm spacing to cover the entire GI tract area. The control algorithm was implemented in Python 3.6, running on a PC (Intel i7-10700F, 32 GB RAM, Win11) and providing real-time visualization of the WCE movement.

To further evaluate the motion characteristics of the WCE, a simulated GI tract environment was created using a custom-designed acrylic plate (made of PMMA). The surface of the acrylic plate included randomly distributed concave and convex features, with each feature spanning approximately 1 to 6 mm in the X-Y plane and heights or depths ranging from 1 to 2 mm. These features were designed to simulate small obstacles or irregularities that the WCE might encounter in real-world environments, providing a test for the system in terms of disturbance resistance and trajectory tracking capabilities. This uneven resistance served as a disturbance, simulating the complex, folded environment of the human GI tract more effectively. A camera was installed at the front of the experimental setup, allowing operators to remotely monitor the WCE movement within the GI tract, facilitating real-time decision-making.

Figure 13b shows the WCE procedure following three different paths through the simulated GI tract environment. WCE was performed using the square trajectory, covering a total path length of 480 mm at a set speed of 10 mm/s, taking 48 s to complete the entire trajectory. WCE was performed using the infinity trajectory, covering a total path length of 580 mm at a set speed of 6.98 mm/s, taking 83 s to complete, which was 30.2% longer than square trajectory, due to the increased complexity of diagonal segments in the inspection path. WCE was performed using the circular trajectory, with a circumference of 377 mm and a set speed of 4.09 mm/s, taking 92 s to complete. This represents a 59.1% increase compared to square trajectory, due to the significantly increased complexity caused by the curved angles in the circular trajectory.

**Figure 14:**
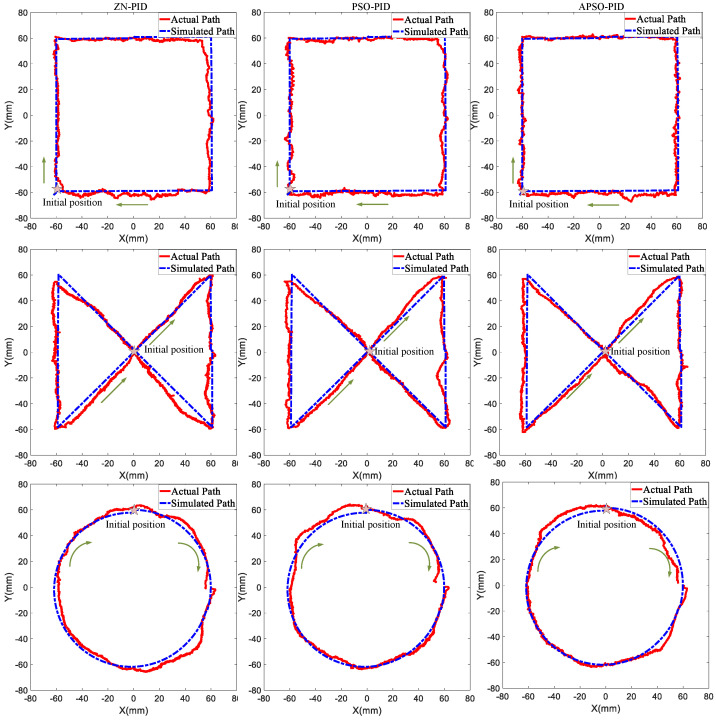
Actual experimental results for the three trajectories.

The results of tracking the three trajectories with different controllers are shown in Figure 14. The red curve represents the actual path of the WCE procedure, while the blue curve shows the simulated path under disturbance. For the square trajectory, the ZN-PID controller exhibited significant jitter at the start and notable deviation and oscillation at the second corner, failing to accurately follow the trajectory, with a noticeable speed change after overcoming resistance. Both the PSO-PID and APSO-PID controllers tracked the trajectory more effectively, though the PSO-PID controller showed a significant speed change after overcoming resistance on the third segment, while the APSO-PID controller maintained a consistent speed throughout and quickly adjusted after passing through the irregular regions.

In the infinity trajectory, all three controllers initially tracked the desired path well. However, as the movement area expanded and disturbances increased, the ZN-PID controller tended to deviate significantly from the desired trajectory and struggled to adjust quickly at corners, resulting in larger deviations and the slowest recovery after overcoming resistance. By contrast, the PSO-PID controller exhibited less deviation than the ZN-PID controller, and its recovery time after disturbances was slower compared to the APSO-PID controller. In the circular trajectory, the ZN-PID controller experienced considerable jitter at the start and generally performed poorly in tracking the trajectory. Tracking with the PSO-PID controller was less smooth compared to the APSO-PID controller, indicating that the APSO-PID controller offers superior stability. Table 5 provides the RMSE and MAE metrics for the three controllers across multiple repeat experiments in tracking the three trajectories.

Table 5 shows that, for the square trajectory, the APSO-PID controller improved accuracy by 0.3 mm and 0.17 mm compared to the ZN-PID and PSO-PID controllers, respectively; for the infinity trajectory, it achieved accuracy improvements of 0.15 mm and 0.07 mm; and for the circular trajectory, it enhanced accuracy by 0.28 mm and 0.13 mm. The tracking error for all three trajectories remained below 4.17 mm, satisfying the basic motion control requirement of less than 5.88 mm, which ensures effective motion control performance [36]. Overall, the APSO-PID controller outperforms the other two controllers, offering shorter adjustment times after disturbances, stronger disturbance rejection, and greater robustness.

## 5. Conclusions

In this work, an actuation device based on external mobile electromagnetic coils was designed and implemented, utilizing a three-axis gantry robot arm and a four-electromagnetic coil module to achieve three degrees of freedom for the WCE within a 250 mm × 250 mm space. The APSO algorithm was used to dynamically optimize the currents in the four coils, ensuring precise turning control during WCE movement. Additionally, each trajectory experiment utilized ZN, PSO, and APSO algorithms to optimize PID parameters, allowing for a comparative evaluation of motion control performance across different optimization methods. In the APSO-PID controller, the APSO algorithm was employed to dynamically optimize the PID parameters, enabling the WCE to precisely track the desired trajectory signals and quickly adjust when subjected to disturbances. Through simulations of the complex GI tract environment and validation in physical experiments, the APSO-PID controller exhibited outstanding dynamic and steady-state performance under both non-disturbance conditions and various external disturbances. Real-world experiments show that, compared to the ZN-PID controller, the APSO-PID controller improved tracking accuracy by 0.3 mm, 0.15 mm, and 0.28 mm on square trajectory, infinity trajectory, and circular trajectory, respectively. Compared to the PSO-PID controller, it improved tracking accuracy by 0.17 mm, 0.07 mm, and 0.13 mm on the same trajectories, confirming its superior control performance in complex tasks.

However, challenges remain in precisely controlling magnetic flux density and multi-degree-of-freedom motion in the current design. Future work will focus on conducting preclinical animal trials to further validate the control performance of this magnetic actuation system and extend this approach to other medical magnetic robots, such as magnetic catheters and magnetic helical robots, enhancing their effectiveness in intelligent inspection, drug delivery, and tissue sampling. Furthermore, we plan to combine sensor data from various medical imaging systems, such as ultrasound imaging and stereoscopic endoscopy, to further enhance the accuracy and robustness of the system.

## Figures and Tables

**Table 1:** Control parameters and specifications.

Item	Specifications
Control parameters	$45\leq K_{p}\leq 75$, $15\leq K_{i}\leq 29$, $0\leq K_{d}\leq 2$
Parameters	Swarm size: m=100; Dimension: D=6; The maximum iteration: $k_{\max}=400$; Learning factors: $c_{1}=c_{2}=2$; The inertia weight increases nonlinearly from: $\omega_{\min}=0.4$ to $\omega_{\max}$ = 0.9 in accordance with Equation (13); $a=0.2k_{\max}$, $b=0.6k_{\max}$, $c=0.9k_{\max}$.

**Table 2:** Performance comparison.

Disturbance Types	Controller	Overshoot ($M_{p}$)	Setting Time ($t_{s}$)	Steady-State Error ($E_{\mathrm{ss}}$)
	ZN-PID	-	0.20259	0.001311
No disturbance	PSO-PID	2.6%	0.088	0.000085
	APSO-PID	0.6%	0.065726	0.000355
	ZN-PID	-	0.28575	0.001408
Composite disturbance	PSO-PID	2.2%	0.10422	0.00098
	APSO-PID	0.6%	0.08061	0.000954
	ZN-PID	-	0.182	0.001059
Random disturbance	PSO-PID	2.33%	0.073	0.00023
	APSO-PID	0.545%	0.07	0.000212

**Table 3:** RMSE and MAE under random disturbance.

Trajectory	Controllers	RMSE (mm)	MAE (mm)
	ZN-PID	0.63	0.69
Square trajectory	PSO-PID	0.49	0.51
	APSO-PID	0.40	0.42
	ZN-PID	0.51	0.58
Infinity trajectory	PSO-PID	0.41	0.45
	APSO-PID	0.32	0.36
	ZN-PID	0.53	0.66
Circular trajectory	PSO-PID	0.40	0.49
	APSO-PID	0.34	0.40

**Table 4:** RMSE and MAE under upward step disturbance.

Trajectory	Controllers	RMSE (mm)	MAE (mm)
	ZN-PID	1.40	1.64
Square trajectory	PSO-PID	1.34	1.58
	APSO-PID	1.27	1.40
	ZN-PID	1.39	1.71
Infinity trajectory	PSO-PID	1.33	1.65
	APSO-PID	1.29	1.61
	ZN-PID	2.30	2.77
Circular trajectory	PSO-PID	2.29	2.73
	APSO-PID	2.27	2.72

**Table 5:** RMSE and MAE in actual experimental results.

Trajectory	Controllers	RMSE (mm)	MAE (mm)
	ZN-PID	2.85	3.10
Square trajectory	PSO-PID	2.68	3.03
	APSO-PID	2.55	2.92
	ZN-PID	4.17	4.46
Infinity trajectory	PSO-PID	4.10	4.39
	APSO-PID	4.02	4.23
	ZN-PID	3.92	4.08
Circular trajectory	PSO-PID	3.79	3.95
	APSO-PID	3.64	3.82

## Data Availability

The original contributions presented in the study are included in the article; further inquiries can be directed to the corresponding authors.
